# Neogene Fallout Tuffs from the Yellowstone Hotspot in the Columbia Plateau Region, Oregon, Washington and Idaho, USA

**DOI:** 10.1371/journal.pone.0044205

**Published:** 2012-10-12

**Authors:** Barbara P. Nash, Michael E. Perkins

**Affiliations:** Department of Geology and Geophysics, University of Utah, Salt Lake City, Utah, United States of America; University of Oxford, United Kingdom

## Abstract

Sedimentary sequences in the Columbia Plateau region of the Pacific Northwest ranging in age from 16–4 Ma contain fallout tuffs whose origins lie in volcanic centers of the Yellowstone hotspot in northwestern Nevada, eastern Oregon and the Snake River Plain in Idaho. Silicic volcanism began in the region contemporaneously with early eruptions of the Columbia River Basalt Group (CRBG), and the abundance of widespread fallout tuffs provides the opportunity to establish a tephrostratigrahic framework for the region. Sedimentary basins with volcaniclastic deposits also contain diverse assemblages of fauna and flora that were preserved during the Mid-Miocene Climatic Optimum, including Sucker Creek, Mascall, Latah, Virgin Valley and Trout Creek. Correlation of ashfall units establish that the lower Bully Creek Formation in eastern Oregon is contemporaneous with the Virgin Valley Formation, the Sucker Creek Formation, Oregon and Idaho, Trout Creek Formation, Oregon, and the Latah Formation in the Clearwater Embayment in Washington and Idaho. In addition, it can be established that the Trout Creek flora are younger than the Mascall and Latah flora. A tentative correlation of a fallout tuff from the Clarkia fossil beds, Idaho, with a pumice bed in the Bully Creek Formation places the remarkably well preserved Clarkia flora assemblage between the Mascall and Trout Creek flora. Large-volume supereruptions that originated between 11.8 and 10.1 Ma from the Bruneau-Jarbidge and Twin Falls volcanic centers of the Yellowstone hotspot in the central Snake River Plain deposited voluminous fallout tuffs in the Ellensberg Formation which forms sedimentary interbeds in the CRBG. These occurrences extend the known distribution of these fallout tuffs 500 km to the northwest of their source in the Snake River Plain. Heretofore, the distal products of these large eruptions had only been recognized to the east of their sources in the High Plains of Nebraska and Kansas.

## Introduction

Commencing at about 16.6 Ma, the initial phase of the Yellowstone hotspot was dominated by the voluminous basaltic volcanism of the Columbia River Basalt Group (CRBG), whereas in the subsequent evolution of the hotspot along the Snake River Plain in Idaho to its present location at Yellowstone National Park, rhyolitic volcanism has been volumetrically dominant over basaltic. Silicic volcanism generated by the hotspot occurred contemporaneously with the eruption of the CRBG, albeit in more limited volume and in some cases from as yet unidentified volcanic centers. In this paper we describe the occurrences of widespread hotspot source rhyolitic tuffs in sedimentary sequences associated in time with the CRBG, those that occur in sedimentary interbeds within the CRBG, as well as a few younger occurrences in the region. A number of these ash fall tuffs are the result of large-volume eruptions, and their widespread distribution permits the correlation of sedimentary units with abundant fossil flora and fauna in geographically separate depositional basins as well as establishing ages for sedimentary sequences associated in space and time with the CRBG. Additionally, documentation of hotspot fallout tuffs in the Pacific Northwest extends the known areal distribution of several large supereruptions in the central Snake River Plain whose products have been identified as far as 1,600 km to the east in the high plains of Nebraska and Kansas.

The Steens Basalt sequence was the first of the flood-basalt eruptions of the Yellowstone hotspot, erupted in southeastern Oregon. The 900 m section of basalt at Steens Mountain was erupted across a reverse (R_o_) to normal (N_o_) magnetostratigraphic transition and has been dated at 16.60±0.02 Ma [1]. Eruptive centers subsequently migrated radially north along the Chief Joseph trend, along the Monument dike swarm towards Picture Gorge, and southward along the Northern Nevada rift (see Camp and Ross [2] for a detailed discussion of the temporal distribution of basaltic volcanism). Basaltic volcanism continued in the Columbia Plateau region to about 6 Ma [3] although 90% of CRBG volcanism occurred between 16.6 and 15 Ma, including the Imnaha and Grand Ronde basalts on the Columbia Plateau. Although most of the literature has focused on basaltic volcanism in the early stages of the hotspot, there is ample evidence for contemporaneous silicic volcanism during this interval. The earliest dated silicic eruptions occurred in northwestern Nevada and southern Oregon in the High Rock volcanic center between 16.5 and 15.4 Ma [4]–[7], the McDermitt caldera complex (16.45–14.6 Ma) [8], the Lake Owyhee volcanic fields of northern Nevada and southeastern Oregon (15.9–14.7 Ma) [9], [10] the Oregon High Plains (15.6–15.3 Ma) [11] and the Oregon-Idaho graben (15.3–10.5 Ma) [12]. Prior to 15.2 Ma, the silicic volcanic centers were situated in the accreted oceanic terrains west of the Precambrian continental boundary as delineated by the ^87^Sr/^86^Sr 0.706 line and are characterized by positive values of ε_Nd_ [13]. By 15.2 Ma the hotspot had encountered the craton as the North American Precambrian lithosphere overrode the deep-seated thermal anomaly, and subsequent eruptions involved magmas derived from the interaction of mantle basalt and Precambrian crust that produced a more focussed and narrow track leading to the development of the Snake River Plain and the modern day Yellowstone Volcanic Plateau.

## Methods

### Sample collection

**Figure 1:**
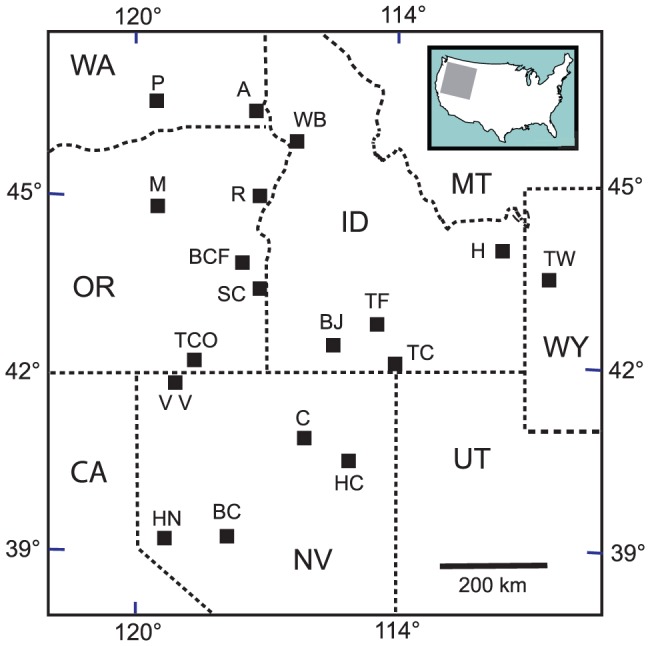
Index map of major localities including reference tephra sections (**Fig. 6**). A = Asotin; BC = Buffalo Canyon; BCF = Bully Creek Fm; BJ = Bruneau-Jarbidge volcanic center; C = Carlin; H = Heise volcanic center; HC = Huntington Creek; HN = Hazen; M = Mascall Fm; P = Pasco Basin; R = Richland, OR; SC = Sucker Creek; TC = Trapper Creek; TCO = Trout Creek; TF = Twin Falls volcanic center; TW = Teewinot Fm; VV = Virgin Valley; WB = White Bird, ID.

Samples of vitric fallout tuffs were collected from sedimentary sequences in Washington, Oregon, Idaho and northeastern Nevada. This study is restricted to the chemical composition of volcanic glass, and, although it may be of interest to paleontolgists and palynologists, the study does not involve the collection or study of fossil animals or plants, nor does the study involve endangered or protected species. No specific permits were required for the described field studies which were conducted on public lands, in many instances along public rights-of-way, although permission to collect on private property was provided for two samples (cpb96-06, cpb09-04). Locations are shown in Figure 1, and information on sample localities is provided in the Appendix S1. Table S1 provides an index to analyzed samples together with available information on ages and neodymium isotopes. A number of occurrences in southern Washington were originally described by Schmincke [14] in his classic study of the flow of invasive basalt into sedimentary interbeds that results in the formation of peperites. Those localities are noted in the Appendix S1.

### Sample preparation

If necessary, the field sample was gently disaggregated with mortar and pestle and then sieved to extract the 60–120 mesh fraction. The sample was washed with distilled water, treated with 10% HNO_3_ to remove any carbonate and then treated briefly with 5% HF to remove clays or other material that may adhere to glass shards. The sample, which normally consisted of 95–99% glass shards, was then mounted in epoxy and polished for electron microprobe analysis.

### Electron microprobe analysis

Analyses were performed with a Cameca SX-50 instrument at the University of Utah using natural and synthetic standards. Approximately 20 glass shards were analyzed for each sample; only a single analysis was performed on each shard. Analytical conditions were 15 keV accelerating voltage, 25nA beam current and a 10–25 µm beam diameter. Oxygen was determined directly such that the analytical total provides a measure of the quality of the analysis together with an estimate of the water content [15]. Because glass shards from Neogene fallout tuffs are hydrated and hydrogen is not determined by microprobe analysis, analytical totals calculated as oxides invariably total less than 100 weight percent, typically 94–96%. This traditional method of reporting analytical results on hydrated glass provides no basis for evaluating the quality of the analysis. This can especially be a concern because of the enhanced potential for migration of Na under the electron beam in hydrated glasses. Individual shard analysis are provided in the Table S4 together with an estimate of water content determined from the difference in measured and stoichiometric oxygen. Sample localities are described in the Appendix S1.

### Correlation of fallout tuffs

**Figure 2:**
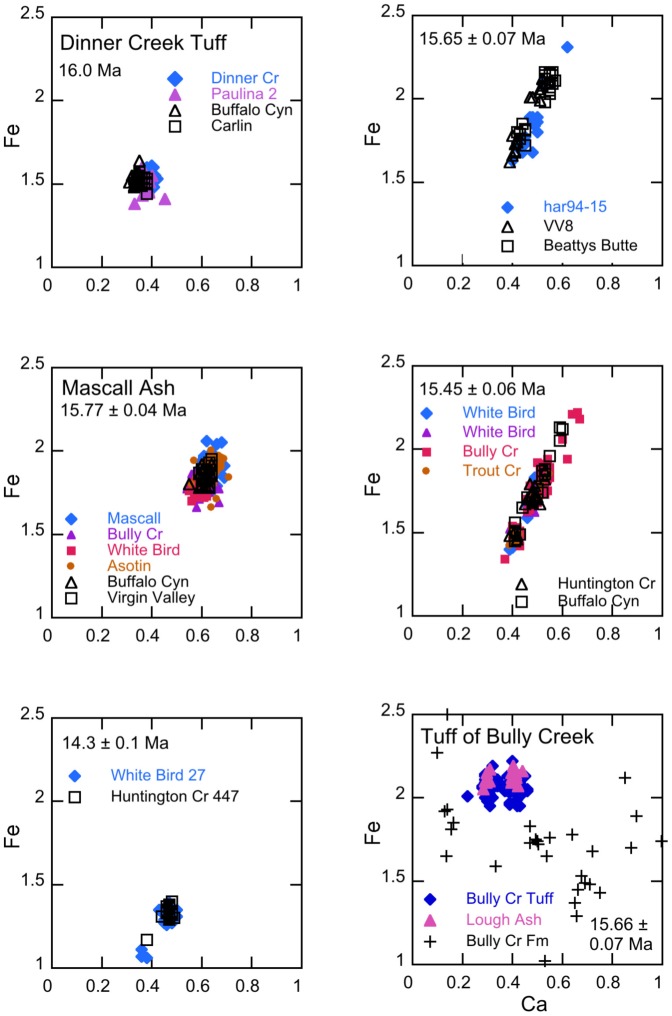
Electron microprobe analyses of Fe and Ca (wt %) in individual glass shards of correlative fallout tuffs, 16–15.5 Ma. Correlative ashes in reference sections are shown in open symbols. In the lower right panel, for comparison with the correlative ash pair, the plus symbols represent average compositions of glass in the other analyzed (and non-correlative) fallout tuffs in the Bully Creek Formation.

Ash units are correlated on the basis of the chemical composition of glass and stratigraphic integrity. First, the average analysis of approximately 20 glass shards from each unknown ash was compared with over 3,000 analyses of glass in western U.S. Neogene tuffs in the University of Utah tephra database. Ashes with similar glass compositions were identified by computing the statistical distance between the average composition of pairs of ashes as described by Perkins et al. [16]. Some elements are not used in comparing ashes, particularly Na and K that are susceptible to variable exchange in hydrated glasses. Potential correlative samples were then compared on an individual shard basis to determine if the distribution of shard populations about the mean value of each was similar. The correlation of fallout tuffs by chemical composition of glass is, of course, only permissive. That is, it is possible that two fallout tuffs from different sources or erupted at different times may have the same chemical composition. Nonetheless, the variation in chemical composition of natural glass is sufficient such that many samples may be ruled as not correlative based on solely on compositional differences. An example of such variability is shown in Fig. 2 in the panel for the Bully Creek Tuff in which a pair of correlative ashes are shown together with the average compositions of all other analyzed ashes in the Bully Creek Formation. In this instance, only the Tuff of Bully Creek is correlative with the Lough ash from another stratigraphic section.

Although mineral abundances in fallout tuffs may change with distance from the source due to preferential winnowing of denser mineral phases, differences in mineral assemblages and the chemical composition of glass make it straightforward to distinguish between tuffs with sources in the Cascade subduction zone and those along the track of the Yellowstone hotspot. With but one exception, the Tuff of Arbon Valley, rhyolites associated with the Yellowstone hotspot do not contain biotite, and hornblende is rare. In contrast, hornblende and biotite are common to dacitic tuffs from Cascade volcanoes, and the distinction between the two sources can often be made in hand specimen in the field.

Work in our lab has demonstrated that it is possible to identify the tectonic regime from which Neogene fallout tuffs in the western U. S. were erupted based solely on chemical composition [17]. Glasses from hotspot tuffs have lower Ca for equivalent Fe contents than glass from Cascadia sources. In general, populations of glass shards in individual hotspot tufs are more restricted in composition or may contain several distinct compositional modes. Glasses from Cascade sources are more dacitic in composition with relatively higher Ca, and they often exhibit a smooth variation over a considerable compositional range as a result of low-pressure crystal fractionation and eruption from continuously zoned magma reservoirs. Both tectonic regimes can be distinguished from Basin and Range sources that have markedly lower contents of Ca and Fe.

## Results

### Early ashfall tuffs erupted during the Mid-Miocene Climatic Optimum

Sedimentary sequences in several depositional basins in the northwestern interior contain rich assemblages of fossil fauna and flora that have been studied extensively, including well-preserved flora from Virgin Valley, Trout Creek, Sucker Creek, Picture Gorge (Mascall Formation), the Latah Formation, and to a lesser extent, the Bully Creek Formation [18]–[22] (see Fig. 1 for locations). These localities and their flora have received renewed interest because it is now recognized that they were deposited during the Mid-Miocene Climatic Optimum [23]–[25] and record environmental conditions different from the late Miocene and Pliocene that reflect a transition to cooler and drier climate climate conditions [26], [27]. The Mid-Miocene Climatic Optimum was also a period of greatly enhanced mammalian diversity [28]. The identification of widespread fallout tuffs, together with published ^40^Ar/^39^Ar ages, allows us to establish the synchroneity of a number of these fossil assemblages as well as to provide a tephrostratigraphic framework for disparate basins across a wide region of active volcanism.

We present new data on early hotspot ashes from localities in Oregon (Malheur Gorge, Picture Gorge and the Bully Creek Formation), western Idaho (Latah Formation) and eastern Washington (Clearwater Embayment) (Figure 1). These localities contains an abundance of fallout tuffs, but in this paper we focus on major fallout tuffs with regional distribution. A number of tuffs in the above localities can be correlated between those localities as well as with tuffs in more distant localities that have previously been described [16], [17] and with recently analyzed tuffs from the Virgin Valley, NV, and Trout Creek, OR. Following a brief overview of the localities, we describe the correlation and distribution of significant tuffs.

#### Bully Creek Formation, southeastern Oregon

The Bully Creek Formation, whose age until now was poorly constrained, outcrops to the northeast of the Malheur Gorge in the vicinity of the communities of Harper and Westfall. The formation consists primarily of tuffaceous sandstone, siltstone and diatomite of fluvial and lacustrine origin. Brooks and O'Brien [29] divided the formation into upper and lower units separated by a distinctive gray massive ash flow tuff that was named the Bully Creek Tuff by Ferns et al. [30]. The upper unit of the formation contains a thin ash flow tuff that bears compositional similarities to the 9.68±0.03 Ma Tuff of Devine Canyon [29], [31], although no direct correlation has been firmly established between the two. The formation is capped by flows of olivine basalt with an ^40^Ar/^39^Ar age of 10.33±1.59 Ma [32]. We have collected and analyzed 49 ash samples from the lower Bully Creek Formation, four of which can be correlated to dated units elsewhere in Oregon, Nevada, Idaho and Washington, and indicate that deposition of the lower half of the formation occurred between approximately 15.8 and 15.5 Ma, just prior to the initial development of the Oregon-Idaho graben immediately to the southeast [12].

#### Latah Formation at White Bird, ID

The Latah Formation, widespread in eastern Washington and western Idaho, consists of fluvial and lacustrine deposits interbedded with CRBG flows, and is known for its well-preserved flora [33], [34]. Fallout tuff bearing beds of the Latah Formation at White Bird, Idaho, are contained within ancient and active landslide deposits; their original stratigraphy has been significantly disrupted. The Formation is underlain by Imnaha basalt and Grande Ronde basalt of magnetostratigraphic units R_1_ and N_1_ [35], but younger Grand Ronde units are absent in the immediate vicinity. At White Bird, where samples were collected (Fig. 1), the Latah Formation is overlain by the Grangeville Member of the of the Saddle Mountain Basalt [35].

#### Virgin Valley and Trout Creek Formations

The Virgin Valley Formation in northwestern Nevada consists of airfall and ashflow tuffs overlain by a thick sequence of fluvial and lacustrine tuffaceous sediments, fallout tuffs, and diatomites that accumulated in the topographic depression of the Virgin Valley caldera that formed in response to the eruption of the Idaho Canyon Tuff dated at 16.30±0.06 Ma. The sediments of the Virgin Valley Formation overlie the post-caldera Canyon Rhyolite that has ^40^Ar/^39^Ar dates of 16.15±0.07 and 16.11±0.03 Ma [36]. The Formation is well known for its Barstovian mammalian fauna [18]. We have collected and analyzed glass from 60 fallout tuffs in the Virgin Valley Formation, many of which are local in origin, but several of which have widespread distribution. Six fallout tuffs in the upper part of the Formation correlate with tuffs in the Trout Creek Formation, outcropping 75 km to the northeast in Oregon. The Trout Creek Formation, whose age is poorly constrained, consists of diatomite, silty sandstone and shales with abundant fallout tuffs, and is known for its well-preserved flora [25]. We have collected and analyzed glass from 24 fallout tuffs in the Trout Creek Formation. One of these tuffs is widespread in the western U.S; five others can be correlated with fallout tuffs in the upper portion of the nearby Virgin Valley Formation.

### Regional correlative fallout tuffs: 16–14 Ma

#### Dinner Creek Tuff

As described above, there are numerous silicic centers in eastern Oregon with associated ash flow tuffs. Attention is focused here on the Dinner Creek Tuff because correlative fallout tuffs can be identified in several distal sedimentary basins in the western interior that establish its widespread distribution and provide constraints on its age. The Dinner Creek Tuff outcrops extensively in the Malheur Gorge as a densely welded ashflow deposit where it lies conformably on the basalt of Malheur Gorge which has been divided into three units [32], [37], [38]. Lower flows in the Malheur Gorge (Lower Pole Creek flows) share chemical and petrographic similarities with the lower flows of the Steens Basalt sequence at Steens Mountain. Basalts intermediate in the section (Upper Pole Creek flows) are similar in composition to Imnaha Basalt of the CRBG, and uppermost lavas of the Birch Creek unit are chemically and petrographically equivalent to basalt of the Grande Ronde Formation of the CRBG. Two ^40^Ar/^39^Ar dates by Swisher et al. [39] of 16.60±0.02 Ma and 16.59±0.02 Ma at the base and top of the Steens Mountain sequence, respectively, indicate that it was erupted within a short interval. Hooper et al. [38] report mean ^40^Ar/^39^Ar ages of 16.9±0.8 Ma for Lower Pole Creek flows, 16.5±0.3 Ma for Upper Pole Creek flows, and 15.7±0.1 for Birch Creek flows. The Dinner Creek Tuff is overlain by flows of the Hunter Creek basalt [39]; the two units may have been erupted simultaneously [40], [41].

Camp et al. [39] provide a whole rock analysis of the Dinner Creek Tuff that compares well with analyses of glass from the base of the Dinner Creek Tuff presented here (Table S2). The fallout tuff has a unimodal shard population with moderate Fe content and relatively low Ca (Fig. 2). Streck et al. [42] estimate that the ash flow covered approximately 20,000 km^2^, and correlative fallout tuff units confirm the widespread distribution of the fallout tuff from central Oregon to central Nevada. The chemical composition of glass from the base of the Dinner Creek ashflow tuff in Malheur Gorge is correlative with a fallout tuff in the Paulina basin 165 km to the west (sample mas96-02, Table S1), as well as a fallout ash located low in the stratigraphic section at Buffalo Canyon in central Nevada [16] that has a stratigraphically interpolated age of 16.0±0.1 Ma, and a fallout tuff exposed in the Carlin basin in northern Nevada that has an ^40^Ar/^39^Ar age of 16.30±0.25 [43]. Additional ages for the Dinner Creek Tuff include a K/Ar age of 15.3±0.4 Ma and two ^40^Ar/^39^Ar ages of 15.4±0.6 and 15.1±2.0 Ma [38], [44]. The Hunter Creek Basalt, which overlies the Dinner Creek Tuff, has reported ages of 15.89±0.26 and 15.4±0.67 Ma [12], [32]. Streck et al. [42] have identified the source of the Dinner Creek Tuff as a volcanic center between Castle Rock and Ironside Mountain, Oregon, that produced multiple ignimbrites and ashfall tuffs that constitute what they describe as the Dinner Creek Tuff assemblage and range in age from 15.9±0.13 to 15.4±0.16. They suggest that this same center is the source of the Mascall Tuff (see below). We recognize a single fallout tuff layer in the Buffalo Canyon, NV, section that we correlate with the Dinner Creek Tuff exposed along the Malheur Gorge. The Dinner Creek Tuff lies stratigraphically below the Mascall Tuff and Virgin Valley 8 fallout tuff, and based on the age determinations of a correlative tuff (16.30±0.25 Ma), a stratigraphic age at Buffalo Canyon (16.0±0.1 Ma), and the older of the ages for the Dinner Creek Tuff [42], we favor an age of 16.0±0.1 Ma for the regionally distributed ash of the Dinner Creek Tuff ignimbrite that is exposed in the Malheur Gorge.

#### Mascall Tuff

The Mascall Tuff is one of the most widespread of the early hotspot tuffs, ocurring in localities in Nevada, Oregon, Washington and Idaho. The Mascall fallout tuff is exposed as a prominent 1.5 m thick bed in the type Mascall Formation near Picture Gorge, Oregon, and has an ^40^Ar/^39^Ar age of 15.77±0.04 Ma [45]. The Formation overlies the Picture Gorge basalt that was erupted across the N_1_R_2_ magnetostratigraphic boundary at ∼16.1 Ma [46]. The compositionally unimodal fallout tuff (Fig. 2) is widely distributed, occurring low in the Bully Creek Formation in southeastern Oregon and in the Latah Formation at White Bird, Idaho, as well as other Miocene sedimentary sequences at Virgin Valley in northwestern Nevada, Huntington Creek, central Nevada, and Sucker Creek, eastern Oregon. The fallout tuff in the type section shows no vertical zonation in composition as shown by analyses of glass from the base, and 25 and 100 cm above the base (Table S4). Streck et al. [42] suggest that the Mascall Tuff and Dinner Creek Tuff were erupted from the same volcanic center that evolved to more mafic compositions with time, consistent with the higher Fe and Ca contents of the younger Mascall ash. Although differing in Fe and Ca contents, the two units share some similarities including relatively high Ba (Table S2). A conspicuous 1.2 meter thick fallout tuff that is compositionally indistinguishable occurs in a sedimentary interbed in the Clearwater Embayment at Asotin grade, Washington (Fig. 1). The interbed containing the fallout tuff is mapped as “sedimentary interbed 1”, lying above flows of the Priest Rapids member of the CRBG that has an age of 14.5 Ma [47]. However, the probable correlation with the Mascall Tuff and the fallout tuff of similar age in the Latah Formation at White Bird suggest that the Asotin interbed may be older than previously thought and is time-equivalent to the Vantage Member of the Ellensburg Formation [48].

#### Virgin Valley fallout tuffs

Of approximately 60 individual fallout tuffs that we have analyzed from the Virgin Valley, 10 have regional distribution, with two occurring in the Bully Creek Formation. Virgin Valley 8, a widespread and distinctive polymodal tuff (Fig. 2) known locally in the Virgin Valley as the “blue sand”, occurs below the Mascall fallout tuff in the Bully Creek Formation and is compositionally indistinguishable from the fallout tuff associated with the tuff of Beatys Butte in southern Oregon, 60 km north of Virgin Valley. Dawson [49] concluded that the Barstovian mammalian fauna at Beatys Butte described initially by Wallace [50] is contemporaneous with the Mascall and Sucker Creek faunas. The Virgin Valley 8 fallout tuff is also found in several other localities in the western U.S., including the Tesuque Formation 1,300 km to the southeast in the Rio Grande Valley, NM, Sucker Creek, OR, and Buffalo Canyon, NV, where it has a stratigraphic age of 15.4**±**0.25 Ma [16], [17]. The tuff of Beatty Butte has a ^40^Ar/^39^Ar age of 15.65±0.07 Ma [45]. A stratigraphically younger undated fallout tuff at Virgin Valley (vvy94-57) also occurs in the Bully Creek Formation above Virgin Valley 8.

#### Huntington Creek 1

A pair of fallout tuffs occur in stratigraphic succession at White Bird and Bully Creek. The lower tuff (samples cpb00-28 and har94-21, respectively) is undated, has not been found elsewhere, and has a distinctive composition characterized by high Ca and relatively low Fe (Fig. 2). The upper tuff of the pair (har94-22b) is a 30 cm thick gray vitric tuff that is correlative with the Huntington Creek 1 fallout tuff in east-central Nevada [16]. This widespread tri-modal tuff (Fig. 2) lies immediately below the Tuff of Bully Creek. One especially conspicuous outcrop of the fallout tuff is approximately half way up a prominent pinnacle on the southwest side of the Harper-Westfall road at Danger Point. The fallout tuff is also present in Nevada at Buffalo Canyon and Carlin, and at Trout Creek, Oregon. It has a ^40^Ar/^39^Ar age of 15.35±0.06 Ma [16], [43].

#### Tuff of Bully Creek

The Tuff of Bully Creek is a prominent 15 m thick gray, vitric, non-welded to partially-welded ash flow tuff situated near the middle of the Bully Creek Formation [29] [30]. The glass shards are distinctly bimodal in composition and characterized by high Fe and Mn and low Ca (Fig. 2); the tuff is correlative with the Lough fallout tuff that lies near the base of the Sucker Creek Formation 60 km to the southeast. The tuff at Sucker Creek has a ^40^Ar/^39^Ar age of 15.66±0.07 [51]. The Tuff of Bully Creek lies immediately above the Huntington Creek 1 fallout tuff that is locally partially thermally altered by the overlying ash flow, consistent with overlapping uncertainties in the ^40^Ar/^39^Ar ages for the two units. The occurrence of the Huntington Creek 1 fallout tuff at White Bird, and its close proximity to the Tuff of Bully Creek establishes that the Sucker Creek flora assemblage in the mid-Miocene Sucker Creek Formation [22], [52] is contemporaneous with the Mascall flora and the previously undated fossil flora in the Latah Formation at White Bird, Idaho [53].

#### Clarkia fossil beds, northern Idaho

A short-lived Miocene lake, formed by the damming of the ancestral St. Maries river, deposited ∼12 m of laminated clays that contains several volcanic fallout tuff layers. The strata are reknowned for their diverse and exceptionally well-preserved fossil flora [54]–[56]. To our knowledge, the volcanic tuffs have not been dated radiometrically. The lowermost gray vitric tuff at site P-33 (lithounit 2c of Smiley and Rember [54]) is 50 cm thick, coarser towards the massive base and laminated in the upper 25 cm. It is characterized by both high Fe and Ca, and the only correlative unit in our database is a 2 m thick pumiceous bed (har02-48) that lies a few meters below the Tuff of Bully Creek at a single locality in the Bully Creek Formation. If this correlation is valid, it places the age of the Clarkia fossil beds at approximately 15.7 Ma. At site P-33, the distinctive 10 cm thick white vitric fallout tuff (lithounit 4 of Smiley and Rember [54]) that lies 2 m above the lower tuff is unfortunately not recognized elsewhere in our tephra database.

#### Obliterator fallout tuff

**Figure 3:**
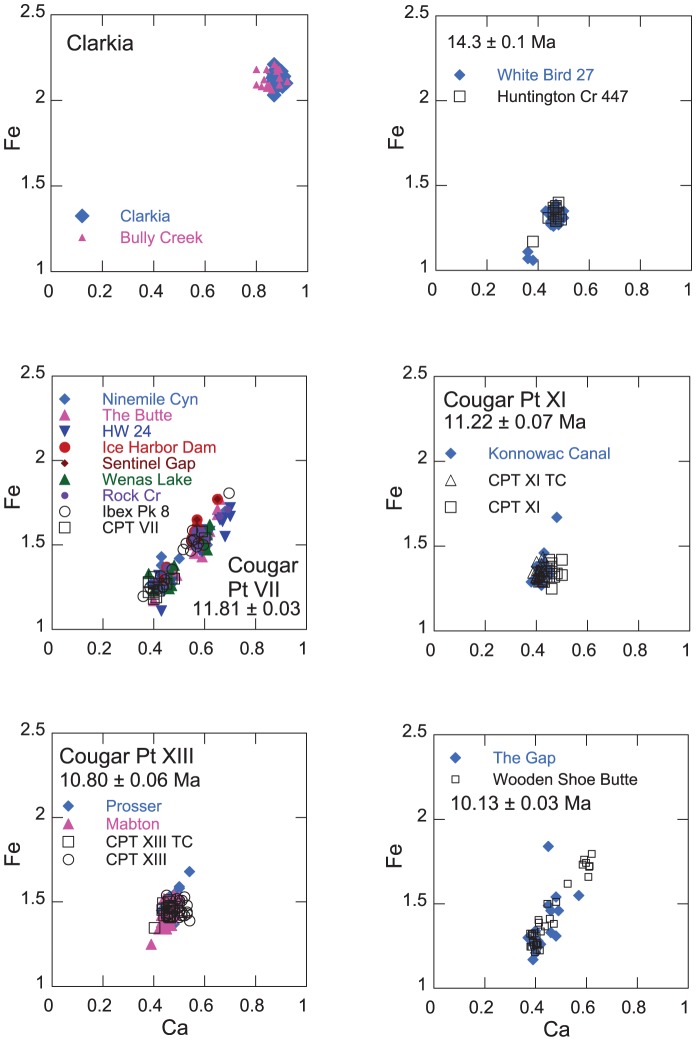
Electron microprobe analyses of Fe and Ca (wt %) in individual glass shards of correlative fallout tuffs, 15.5–10 Ma. Correlative ashes in reference sections are shown in open symbols.

A correlative to the unimodal Obliterator ash (Fig. 3) occurs in the Payette Formation in the Weiser Embayment in western Idaho, approximately 70 m above the basalt of Linson Gulch in the Holland Gulch Quadrangle [57]. The Obliterator tuff, which also occurs at Sucker Creek, OR, and Huntington Creek, NV, has an ^40^Ar/^39^Ar age of 14.93±0.08 Ma [16] and is thus contemporaneous with the Latah Formation at White Bird.

#### White Bird 27

The youngest fallout tuff analyzed from the Latah Formation at White Bird, ID, is compositionally bimodal (Fig. 3) and correlative with a tuff at Huntington Creek, NV, where it has a stratigraphically interpolated age of 14.3±0.1 Ma [16].

### Fallout tuffs from the Bruneau-Jarbidge eruptive center, Snake River Plain, ID, 12.8–10.5 Ma

The influx of sediment from highlands both to the west and east of the Columbia Plateau, together with the disruption of local drainages by basalt flows, resulted in numerous sedimentary interbeds within the CRBG. Prominent sedimentary interbeds occur in the Clearwater and Weiser Embayments near the Washington-Oregon-Idaho border and in the Ellensburg Formation in the Pasco Basin on the western margin of the Columbia River Plateau. The Ellensburg Formation contains abundant deposits of volcaniclastic debris derived from ancestral volcanoes of the adjacent Cascade Range [48], [58]–[60]. Sediments in the Ellensburg Formation occur mostly as air-fall tuffs, reworked fluvial sediments and debris flows; volcanic material consists predominantly of hornblende-biotite dacite, common to explosive volcanism in the Cascade Range. Fallout tuffs containing hornblende with or without biotite tend to be relatively thin, often contain pumice clasts, and are consistent with moderate-sized Plinian eruptions from relatively nearby sources [48]. However, there are occasional thicker (1–8 m) gray vitric tuffs that are crystal-poor and devoid of hornblende and biotite, a number of which were originally described by Schmincke [14] in his study of peperites in the region. As shown below, these fallout tuffs were not derived from Miocene Cascade volcanoes, but rather have their origin in large volume eruptions originating from volcanic centers of the Yellowstone hotspot 500 km to the southeast in the Snake River Plain in south-central Idaho. Because of their widespread distribution, distinctive chemical fingerprints, and established eruption ages, these ashfall units serve as useful stratigraphic markers and can aid in correlating discontinuous outcrops of sedimentary sequences associated with the Columbia River Basalt Group. Chemical analyses of glass from correlated ashes are provided in Table S3.

The Bruneau-Jarbidge volcanic center in the central Snake River Plain was active from 12.8 to 8 Ma and erupted approximately 10^4^ km^3^ of rhyolite [17], [61]. This interval in the eruptive history of the Yellowstone hotspot was distinguished by higher magmatic temperatures and eruption frequency than experienced by silicic volcanism at the younger Heise and Yellowstone Volcanic Plateau eruptive centers [17]. At the Bruneau-Jarbidge volcanic center, the explosive episode is represented by the Cougar Point Tuff (CPT) that consists of ten voluminous ashflow tuff members erupted between 12.8 and ∼10.5 Ma that are exposed in deeply cut canyons along the Idaho-Nevada border on the southern margin of the Snake River Plain. In some areas such as the Black Rock Escarpment on the Bruneau River, the composite section reaches a thickness of ∼475 m [62]. Fallout tuffs from members of the CPT have subcontinental distribution with outcroppings as far as 1500 km to the east in the central High Plains of Nebraska and Kansas [17]. The occurences described here are the first from the Pacific Northwest, and they considerably extend the known distribution of fallout tuffs from these supereruptions. Eleven occurrences of fallout tuffs in southern Washington and adjacent Oregon are correlative with three members of the Cougar Point Tuff (Fig. 3).

#### Cougar Point Tuff member VII - Ibex Peak 8 tuff

**Figure 4:**
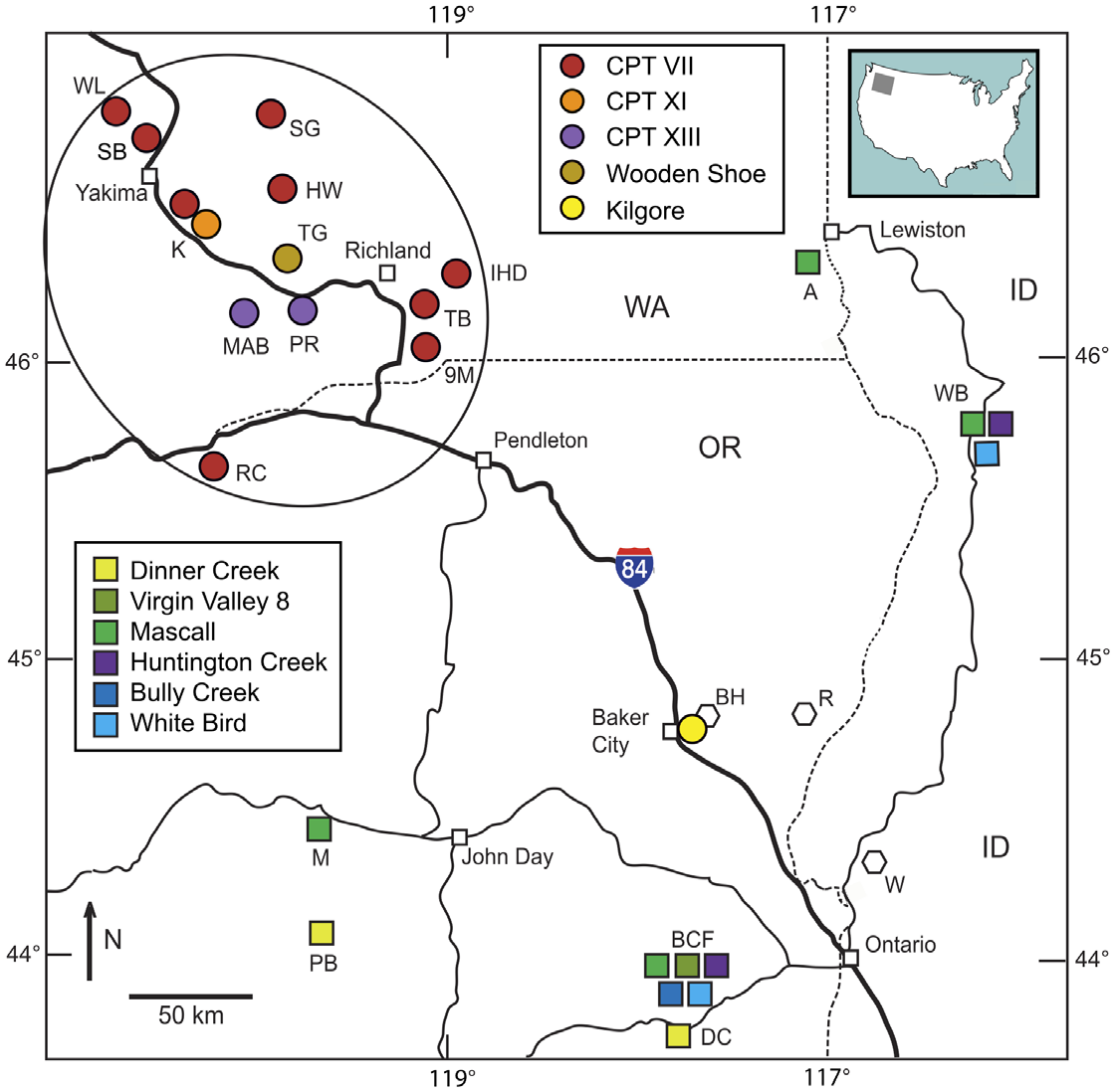
Index map for analyzed fallout tuff samples. Tuffs between 16 and 15 Ma erupted from volcanic sources in Oregon and northern Nevada are shown in square symbols. Notations: A = Asotin; BCF = Bully Creek Fm.; BH = Baker-Copperfield Highway; M = Mascall Fm.; MG = Malheur Gorge; PB = Paulina Basin; R = Richland; W = Weiser; WB =  White Bird. Younger tuffs with sources in the Bruneau-Jarbidge, Twin Falls and Heise volcanic centers on the Snake River Plain are shown in round symbols: 9M = Ninemile Canyon; HW = Highway 24; IHD = Ice Harbor Dam; K = Konnowac Pass and Konnowac Canal; MAB = Mabton; PR = Prosser; RC = Rock Creek; SB = Selah Butte; SG = Sentinel Gap; TB = The Butte; TG = The Gap; WL = Wenas Lake. Oval encloses the extent of exposures of fallout tuffs from members VII, XI and XII of the Cougar Point Tuff.

Fallout tuffs correlative with the distinctive tri-modal Ibex Peak 8 tuff of Trapper Creek, ID (11.80 Ma) have been found in nine localities in the Yakima-Tri Cities area of southern Washington (Fig. 4). The distribution of the unit from Wenas Lake to Ice Harbor Dam, a distance of 150 km, suggests the presence of an extensive depositional basin or system of basins in the region at 11.8 Ma. The Ibex Peak 8 tuff shares many compositional and stratigraphic similarities with member VII of the Cougar Point Tuff that we consider to be the ashflow equivalent. Outcrops of the Ibex Peak 8 tuff in the Yakima fold belt and Pasco Basin range in thickness from 2–7 m. The tuff is reworked in all exposures, and at several localities the ash layer is overlain by basalt. At The Butte near Finley, a 2 m thick exposure of ash (cpb98-13) is irregularly invaded by basalt. The tuff in Ninemile Canyon is >3.5 m thick; the base is not exposed, and it is overlain by vesicular basalt with a 5 cm fused glass layer below the base of basalt (Schmincke, locality 31 [14]). The 3 m thick vitric ash at Highway 24 (ell99-03) is sandwiched between basalt flows, and the exposure of ash at the summit of Konnowac Pass (ell99-05) is overlain by basalt. Schmincke describes in detail the invasion of ash at Ice Harbor dam by the Pomona Basalt (Schmincke, Plate 4, Fig. 3 [14]), and the ^40^Ar/^39^Ar age of 11.81±0.03 Ma on the correlative Tuff of Ibex Peak provides a maximum age for the Pomona Basalt whose age is commonly given as 12 Ma [63]. The position of CPT VII immediately below the Pomona Basalt places the tuff at the top of the Selah Member of the Ellensburg Formation (Smith, Fig. 2 [48]). At Selah Butte, the CPT VII fallout tuff lies at the top of an approximately 40 m thick sedimentary interbed and almost certainly is the ”distinctive vitric tuff” that Smith [58] describes as occurring at the top of the Selah Member. The distribution of the CPT VII tuff indicates the presence of an extensive lacustrine system responsible for the the deposition of the Selah Member. Outcrops of CPT VII at Wenas Lake, Selah Butte and Konnowac Pass define the course of the ancestral Yakima River while more easterly outcrops at Sentinal Gap and Highway 24 locate the position of the ancestral Columbia River [58]. These outcrops, together with those east of Richland and at Rock Creek, OR, indicate that the location of the ancestral Columbia River at about 12 Ma is very nearly its present-day location with the exception of flowing more directly south from Sentinal Gap as evidenced by deposition of the younger CPT XI and XIII tuffs (Fig. 4).

At Idaho City, ID, a 1 m thick ash lies near the base of a 12 m section of fluvial-lacustrine sediments that overlie a normally magnetized Miocene basalt flow [64], [65]. We had previously correlated the Idaho City ash with the ash of Logan Ranch [64]. However, the close proximity in age and the similarity in composition of the ash of Logan Ranch to Cougar Point VII leads us to suspect that the two are either the same unit or were erupted closely in time from the Bruneau-Jarbidge eruptive center.

#### Cougar Point Tuff member XI

An ash poorly exposed in a canal bank at Konnowac Pass (ell99-04) is correlative with Cougar Point XI (11.22±0.07 Ma [61]) (Fig. 3) which was one of the more voluminous eruptions from the Bruneau-Jarbidge center and occurs in numerous localities across the western U. S. and in the High Plains of Nebraska [17]. Its age places it in the Rattlesnake Member of the Ellensburg Formation [48]. The occurrence of CPT XI on the Columbia Plateau extends its distribution well to the northwest of its source in the southwestern Snake River Plain.

#### Cougar Point Tuff, member XIII

The fallout tuffs occurring at Prosser (cpb98-15) and the Mabton-Bickelton road (cpb98-18) are correlative with member XIII of the Cougar Point Tuff (10.75±0.07 Ma [61]) (Fig. 3), another large volume eruption from the Bruneau-Jarbidge center with widespread distribution. The ash at Prosser consists of 12 cm of airfall tuff overlain by 8 m of reworked ash and sediment. The ash is overlain by basalt that has produced a 1 m thick baked zone in the ash immediately below the basalt contact. Its age places it also in the Rattlesnake Member of the Ellensburg Formation [48].

### Hotspot ashes <10.5 Ma

#### Tuff of Wooden Shoe Butte

**Figure 5:**
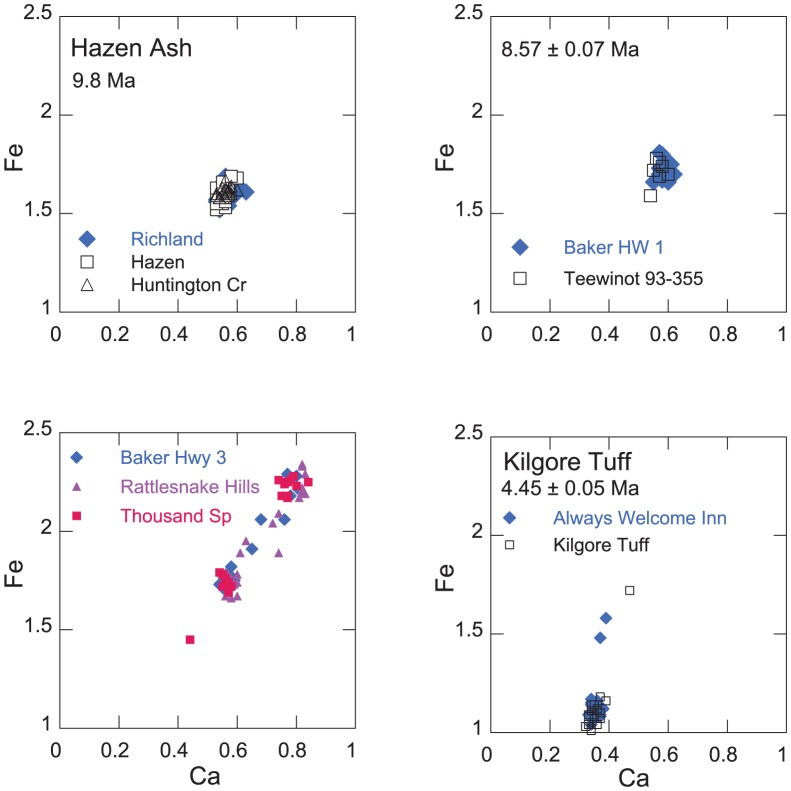
Electron microprobe analyses of Fe and Ca (wt %) in individual glass shards of correlative fallout tuffs, 10–4 Ma. Correlative ashes in reference sections are shown in open symbols.

The fallluot tuff at The Gap is correlative with the Tuff of Wooden Shoe Butte (10.13±0.03 Ma), erupted from the Twin Falls eruptive center in the central Snake River Plain (Fig. 5). Correlative exposures of this tuff occur in the Cassia Mountains of southern Idaho [66], [67]. The exposure of gray vitric tuff at The Gap (Schmincke locality no. 27 [14]) is overlain by basalt, and with its base not exposed, has a minimum thickness of 4 m. Its age places it in the upper Ellensburg Formation, younger than the Elephant Mountain basalt member [48].

#### Unnamed ash at Richland, OR (or99-01)

A 4 m thick gray massive fallout tuff is exposed as a channel deposit above basalt flows of unknown age in a roadcut on highway 89, 1.5 km north of Richland, OR. The unimodal ash is chemically indistinguishable from the 9.8 Ma Hazen ash in eastern Nevada (Fig. 5) [16]–[17].

#### Unnamed ashes, Baker-Copperfield Highway, OR (cpb09-01; cpb09-03)

Two ashes outcrop in close proximity along the Baker-Copperfield Highway in eastern Oregon [68]. Sample cpb09-01 is from Section #1 of Ledgerwood and Van Tassel [64] who report an average ^40^Ar/^39^Ar isochron age of 8.57±0.07 Ma. Because of the similarity to the age of the Prater Creek ashflow tuff erupted from the region of the Malheur basin in central Oregon at 8.48±0.05 Ma, they suggest that it is the source of the Baker-Copperfield ash. However, both ashes outcropping on the Baker-Copperfield highway are chemically distinct from the Prater Creek ash and share affinities with ashes derived from the Twin Falls volcanic center on the Snake River Plain. We correlate the ash from section #1 of Ledgerwood and Van Tassell [68] with a fallout tuff that occurs in the Teewinot Formation in Wyoming that has an interpolated age of ∼8.6 Ma, consistent with the ^40^Ar/^39^Ar age for the Oregon ash. We correlate the ash from section #2 of Ledgerwood and Van Tassell [68] (cpb09-03) with an undated, chemicaly distinct polymodal ash (ell99-06) that occurs in the Rattlesnake Hills in the Pasco Basin, WA, and in the Thousand Spring Formation in northern Nevada (Fig. 5).

#### Ash at the Always Welcome Inn, OR (cpb-09-04-I)

A pale gray friable ash occurs at 6.6 m in a 10 m section of lacustrine and fluvial sediments at the Always Welcome Inn, Baker, Oregon. The early Pliocene (Blancan) section contains a rich and diverse fossil assemblage described by Van Tassell et al. [69] who suggested a preliminary age range for the ash of ∼13–15 Ma with an average plateau age of 13.6±0.4 Ma, leading to the interpretation that the ash is reworked from older Miocene tuffs in the area. However, based on chemical composition of glass, we correlate the ash with the widely distributed and voluminous (1,800 km^3^) Kilgore Tuff, erupted at 4.45±0.05 Ma from the Heise volcanic center in the eastern Snake River Plain [70] (Fig. 5). This age is consistent with an age estimate of ∼4.8–4.3 Ma based on *Ophiomys* fossils at the Always Welcome Inn fossil site [71]. Furthermore, low Fe and Cl≥0.1% is uncharacteristic of Miocene hot spot ashes older than 8 Ma (Tables S2 and S3; [17]). Figure 5 illustrates that shard populations from both localities also contain occasional shards with higher Ca and Fe.

## Conclusions

For many years the conventional description of the history of silicic volcanism of the Yellowstone hotspot had volcanism initiating at ∼16 Ma at the McDermitt caldera complex in northern Nevada and then proceeding sequentially through the Owyhee-Humboldt, Bruneau-Jarbidge, Twin Falls, Picaboo (now discarded), Heise and present-day Yellowstone Plateau volcanic centers [72], [73]. The narrative, with McDermitt as the origin point, was motivated in part by the temporal sequence and approximate linearity of the volcanic centers along an azimuth antiparallel to the North American plate motion. This interpretation did not incorporate information from studies on early volcanism in other localities in the region to the north and west of McDermitt [4]–[6], [9] that did not fall within the narrow hotspot track framework. Further evidence for widespread, abundant volcanism in centers in southeastern Oregon and northern Nevada between 16 and 15 Ma is provided by the abundance of fallout tuffs in both proximal and distal sedimentary basins. As mentioned above, we have identified a total of more than 130 fallout tuffs in the Bully Creek, Virgin Valley, and Trout Creek localities. The majority of these tuffs appear to be from relatively small-volume eruptions because they are not regionally distributed in the manner of the lesser number of large-volume fallout tuffs discussed in this paper. Previously we had similarly observed that over the entire 0–16 myr history of the Yellowstone hotspot, the frequency of silicic eruptions was highest in the 15–16 Ma time interval as recorded in distal stratigraphic sections [17].

Widespread and abundant volcanism is consistent with the hotspot model as developed by Camp and Ross [2] with the arrival of a mantle plume with a plume head initially impinging in the region of present-day southeastern Oregon and expanding radially, resulting in the voluminous outpourings of flood basalts of the Columbia River Group (CRG). The majority of the flood basalt eruptions on the Oregon Plateau occurred between ∼16.6 and ∼15.3 Ma, the latter half contemporaneous with widespread and frequent silicic volcanism in the region. The transition from broadly distributed volcanism to a more focused hotspot track occurs when the mantle thermal anomaly encounters the Precambrian continental core at ∼15.2 Ma [13], and the previously described linear hotspot narrative applies to the volcanism of the Snake River Plain and the Yellowstone volcanic plateau.

**Figure 6:**
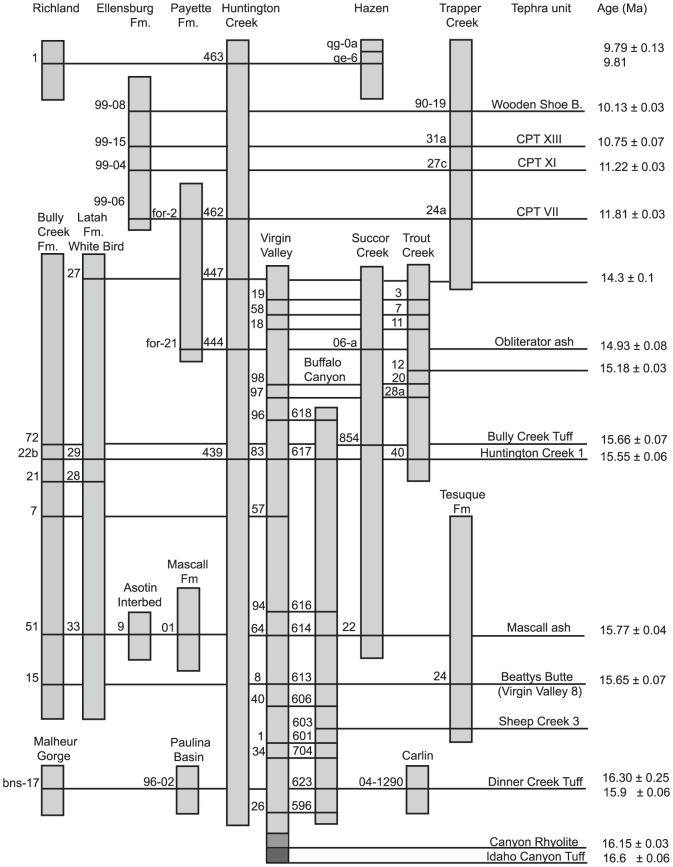
Stratigraphic framework for regional fallout tuffs. Correlation and relative stratigraphic positions of tuffs are shown. No scale is intended for the vertical axis; where known, age dates are provided. Fallout tuffs identified in each section are indicated with a sample number. Correlations between sections are made on the basis of chemical composition of glass shards and consistent stratigraphic position, and are indicated by solid lines. Perkins and coworkers provide detailed sections for Buffalo Canyon, NV, Huntington Creek, NV, and Trapper Creek, ID [16], [17].

Large volume eruptions from multiple volcanic centers produced fallout tuffs distributed across eastern Oregon, southern Washington and western Idaho. Correlative ashes in distal sedimentary sections provide a temporal framework for these early eruptions that range in age from 16.3 to 15 Ma, and a stratigraphic framework for regionally distributed fallout tuffs is provided in Fig. 6. This time interval coincides with the Mid-Miocene Climatic Optimum characterized by an increase in global temperature [23]–[25] as well as enhanced mammalian diversity in the northwestern U.S. [28]. The widespread silicic volcanism in the region resulted in abundant diatomaceous lacustrine deposits [74] accompanied by diverse flora associated with those lacustrine environments – e.g., Virgin Valley, Trout Creek, Sucker Creek, Latah Fm. [18]–[22]. The regional stratigraphic relations determined from correlation of tephra provide some constraints on the relative ages of several of these important flora assemblages.

Eruptions of the Idaho Canyon Tuff and Canyon Rhyolite in the Virgin Valley region of northwestern Nevada between 16.3 and 16.1 Ma were followed closely by the voluminous 16 Ma Dinner Creek Tuff, erupted from a center in eastern Oregon. These predate the Bully Creek Formation. The lower half of the diatomite-rich Bully Creek Formation contains five widely distributed fallout tuffs that place the time of its deposition between 15.8 and 15.5 Ma, contemporaneous with the Mascall and Sucker Creek flora in Oregon and the previously undated Latah flora at White Bird, ID. The Trout Creek flora are contemporaneous with the upper part of the Virgin Valley sequence and are younger than the Mascall and Sucker Creek flora. If the tentative correlation of the Clarkia beds with a unit just underlying the Tuff of Bully Creek is correct, then the Clarkia flora lie stratigraphically above the Mascall flora and below the Trout Creek flora.

Two large-volume units, the Dinner Creek Tuff (16.0 Ma) and the Mascall ash (15.8 Ma), which have positive ε_Nd_ values, as well as the Tuff of Beattys Butte, were erupted from centers in the accreted terrains west of the Precambrian craton. Slightly younger Huntington Creek 1 and Bully Creek Tuff have declining ε_Nd_, indicating increasing involvement of Precambrian crust, transitional to tuffs younger than 15.0 Ma that were erupted from centers entirely underlain by Precambrian crust along the Snake River Plain [13].

**Figure 7:**
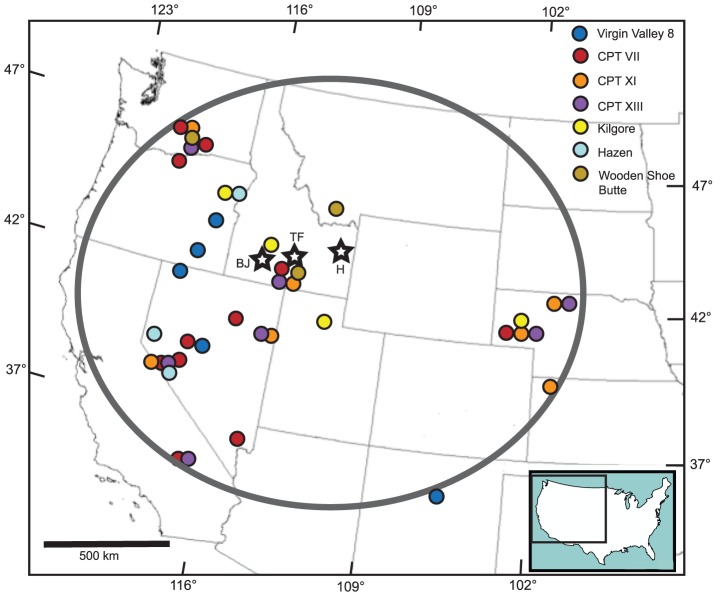
Bruneau-Jarbidge (BJ) Twin Falls (TF) and Heise (H) eruptive centers (open stars) along the track of the Yellowstone hotspot and locations of Cougar Point Tuff members VII, XI and XIII, Tuff of Wooden Shoe Butte, Hazen ash and the Tuff of Kilgore. The occurrences in Washington and Oregon of the products of these supereruptions are newly recognized and significantly extend the known areal distributions of these four supereruptions. The oval encompasses the known distribution for the three members of the Cougar Point Tuff. Also shown are known locations of the widely distributed older Virgin Valley 8 tuff.

The several extensive volcanic centers located along the Snake River Plain and on the Yellowstone volcanic plateau have been the sources of numerous large-volume “supereruptions” with tephra dispersed on a sub-continental scale. Heretofore, the products of these eruptions had only been recognized to the east of their sources, in the central high plains of the U. S. and south to the Gulf of Mexico. The two exceptions being the 2.06 Ma Huckleberry Ridge Tuff and 0.6 Ma Lava Creek B Tuff, whose fallout has been identified well to the west of their Yellowstone source in cores from the Pacific Ocean off the coast of California [75]. The results of this study document the presence of fallout tuffs in the Pacific Northwest from four large-volume eruptions between 11.8 – 10.1 Ma from the Bruneau-Jarbidge and Twin Falls volcanic centers in the central Snake River Plain. The identification of fallout tuffs in south-central WA derived from sources in the Snake River Plain extends their aerial distribution approximately 500 km to the northwest of previously known outcrops (Fig. 7). Similarly, the regional distribution of the Kilgore Tuff, produced by the 4.45 Ma supereruption (1,800 km^3)^ from the Heise volcanic center in eastern Idaho, has been extended to eastern Oregon. Outcrop thicknesses of distal ash deposits of the CPT tuffs (1.5 to 8 m) are comparable to thicknesses of voluminous Quaternary eruptions such as the Lava Creek B Tuff (1000 km^3^) and Huckleberry Ridge Tuff (2500 km^3^) with sources in the Yellowstone Plateau at comparable distances [17] confirming that CPT VII, XI and XIII clearly fall within the supereruption category. However, precise eruption volumes are difficult to assess because original airfall thicknesses are often obscure, and ash thicknesses have been significantly amplified by reworking of primary fall deposits and redeposition in local basins.

## Supporting Information

Appendix S1 **Sample descriptions and locations.**
(DOC)

Table S1 **Index to analyzed samples.**
(XLS)

Table S2 **Averages of electron microprobe analyses of glass in 16–14 Ma Yellowstone hotspot fallout tuffs and corelative tephra.**
(XLS)

Table S3 **Averages of electron microprobe analyses of glass in 13–4 Ma Yellowstone hotspot fallout tuffs and correlative tephra.**
(XLS)

Table S4 **Electron microprobe analyses of individual glass shards (1,343 analyses).**
(XLSX)
