# Supplementary material for: Neogene Fallout Tuffs from the Yellowstone Hotspot in the Columbia Plateau Region, Oregon, Washington and Idaho, USA
Source: PLoS One. 2012 Oct 12;7(10):e44205. doi: 10.1371/journal.pone.0044205 (PMC3470548; doi:10.1371/journal.pone.0044205)
Supplement: Appendix S1 — Sample descriptions and locations. (DOC) [file pone.0044205.s001.doc]

Appendix S1: Sample descriptions and locations.

bns01-17. Air fall pumice at the base of the Dinner Creek ashflow tuff exposed in road cut on Hw. 20, Malheur Canyon, OR. 43.76382° N 118.03356° W.

clk96-01. Gray vitric tuff, Clarkia fossil beds site P-33, unit 2c of Smiley and Rember (1985). 50 cm thick, sample taken from base. 46.99152° N, 116.27633° W.

cpb96-06. Gray vitric tuff in pumicite quarry, east side of highway 243 between Wanapum and Priest Rapids dams at Sentinel Gap. Schmincke locality no. 24. 46.77523° N, 119.91043° W.

cpb98-12. Gray vitric tuff. Approximately 13 km up Ninemile Canyon, Finley, WA. Tuff is > 3.5 m thick; base is not exposed. Overlain by vesicular basalt with a 5 cm fused glass layer below base of basalt. Schmincke locality 31; Plate 2, Figure 1. 46.09023° N, 119.07326° W.

cpb98-13. Indurated vitric tuff, 2 m thick, irregularly invaded by basalt. Exposed in a quarry at the west end of The Butte, Finley, WA. Schmincke locality no. 30. 46.12000° N, 119.04362° W.

cpb98-15. Gray vitric tuff in road cut near summit of highway 221 ~ 4.5 km southeast of Prosser, WA. Outcrop consists of 8 m of reworked ash and sediment underlain by 12 cm of airfall tuff. Sedimentary unit is overlain by basalt with ~1 m baked zone below base of basalt. Sample taken from airfall tuff. Schmincke locality no. 5. 46.20497° N, 119.70836° W.

cpb98-18. Gray vitric tuff on Mabton-Bickelton road at the base of Horse Heaven Hills, WA. Sedimentary interbed with basalt below and above. Tuff lies ~20 m above a second vitric tuff. Schmincke locality no. 28. 46.14053° N, 120.02557° W.

cpb00-25. Gray vitric tuff, Rock Creek, OR. The tuff is 7 m thick, extensively reworked and overlain by mudstones capped by conglomerate. Sample collected from friable base. 45.61763° N, 120.22320° W.

cpb00-27. Pale gray fine-grained vitric tuff, ~ 1 m thick. Exposed in road cut on River Road, White Bird, ID. 45.77358° N, 116.29027° W.

cpb00-29, 30. Vitric tuffs in 6 m thick section exposed in road cut on River Road, White Bird, ID, ~ 1.4 km north of cpb00-27. Three tuffs are exposed in the outcrop. Sample 29 is from the base of the 33 cm thick middle tuff. The uppermost tuff is separated from the middle tuff by a 2 cm thick shale layer. Sample 30 is from the coarser airfall base. The top of the outcrop consists of brown and tan laminated sediments. 45.78136° N, 116.27524° W.

cpb00-33. Friable pale gray tuff in road cut on River Road, White Bird, ID, 3.5 km north of sample cpb00-27. Thickness indeterminate. 45.79275° N, 116.26901° W.

cpb09-01. Friable gray ash 0.25 m thick in road cut on Baker-Copperfield Highway in section of diatomaceous silts. Section #1 of Ledgerwood and Van Tassell (2005). 44.83504° N, 117.58367° W.

cpb09-03. Friable gray ash 1.3 m thick in section with diatomaceous silt and sand in road cut on Baker-Copperfield Highway. Ash is reworked with cross-bedding and cross lamination. Section # 2 of Ledgerwood and Van Tassell (2005). 44.83546° N, 117.56451° W.

cpb09-04. Ash at Always Welcome Inn, Baker, OR. Pale brown, friable ash at 6.6 m in 10 m thick sedimentary sequence of diatomites, silts and fine sands. 44.78520° N, 117.80463° W.

cpb09-09, -10. Gray vitric tuff, 1.2 m thick near the top of sedimentary interbed exposed in road cut on the Asotin grade, WA. Lower 25 cm may be airfall, the upper part is reworked. 46.32088° N, 117.05402° W. (cpb09-09 collected from base; cpb09-10 collected 25 cm above base).

ell99-01. Gray vitric tuff exposed in road cut at southeast end of Wenas Lake, WA. Tuff is ~ 2 m thick and underlies a sequence of fluvial sandstones some of which contain biotite-hornblende dacitic pumice up to 3 cm in diameter. 46.81411° N, 120.67035° W.

ell99-03. Gray laminated vitric tuff, ~ 3m thick, sandwiched between basalt in road cut on highway 24, 3.7 km east of intersection with highway 241. Near Schmincke locality no. 8? 46.54732° N, 119.83394°’ W.

ell99-04. Gray vitric tuff. Poor exposure in canal bank adjacent to road between Donald and Moxie north of summit of Konnowac Pass, Rattlesnake Mountains, WA. 46.51188° N, 120.36758° W.

ell99-05. Gray vitric tuff at summit of Konnowac Pass Road between Donald and Moxie, Rattlesnake Mountains, WA. Tuff is > 1 m thick, coarser towards the base, and overlain by basalt. Near Schmincke locality no. 9. 46.50627° N, 120.36425° W.

ell99-06. Gray vitric tuff on the south flank of the Rattlesnake Hills, WA. Tuff is exposed in road cut on northwest side of road. Tuff is 0.5 m thick and lies within a sequence of sandstones. 46.46434° N, 120.32379° W.

ell99-08. Gray vitric tuff >4 m thick at The Gap, WA. Overlain by basalt, base not exposed. Schmincke locality no. 27. 46.31221° N, 119.79319° W.

ell12-01. Gray vitric tuff in sedimentary interbed on Selah Butte, WA. Tuff is exposed in large road cut on north side of I-82. Tuff is of variable thickness from 0.5 to 1 m, and lies at the top of the interbed, overlain by basalt. 46.72666° N, 120.39244° W.

For02-21. Vitric tuff within the fossil leaf and lignite-bearing silty sediments of the lower Payette Formation, Holland Gulch Quadrangle near Weiser, ID. Tuff lies 70 m above reversed-polarity basalt of Linson Gulch.

Forester-2. Gray vitric tuff, 1 m thick, at base of 12 m section of fluvial-lacustrine arkosic sands, silts and tuffaceous clays overlying basalt, 0.5 km east of Idaho City, ID. 43.82718° N, 115.82540° W.

har94-15. Bully Creek Formation. Gray vitric ash, 70 cm thick exposed in road cut on east side of Harper-Westfall road. The ash outcrops 70 m below the base of the Bully Creek Tuff. 43.95060° N, 117.67785° W.

har02-51. Bully Creek Formation. Fine grained pale gray ash, approximately 30 cm thick near base of section. 43.93768° N, 117.67353° W.

har94-07. Bully Creek Formation. Gray ash, 12 cm thick in diatomite sequence. 43.93768° N, 117.67353° W.

har94-21. Bully Creek Formation. Gray ash, 4.5 cm thick doublet separated by thin clay layer. 43.92672° N, 117.65687° W.

har94-22. Bully Creek Formation. Gray vitric ash, 30 cm thick. Lies 7 m above har94-21. Ash is overlain by ~1 m of alternating diatomite and thin altered ash layers baked by overlying ash flow tuff. 43.92672° N, 117.65687° W.

har94-72. Bully Creek Formation. Tuff of Bully Creek. Gray massive ash flow tuff with vitric fallout deposit at the base. The ash flow tuff is well exposed throughout the formation. Typical thickness is 15 m. 43.94855° N, 117.67292° W.

har02-48. Bully Creek Formation. Pumice bed 2m thick below Tuff of Bully Creek. 43.92216° N, 117.62364° W.

Ice Harbor Dam. Gray to tan vitric tuff exposed along the north side of the Ice Harbor Road immediately northwest of Ice Harbor Dam, Snake River, WA. Tuff is ~ 4 m thick and reworked. The locality is ~1.8 km west of Schmincke locality no. 21. 46.25225° N, 118.88832° W.

mas96-01. Mascall ash. Gray vitric tuff, 1.2 m thick, in type section of Mascall Formation below main fossil bed. At this location the Mascall Formation directly overlies the Picture Gorge Basalt. 44.50851° N, 119.63917° W. Also samples cpb09-05 (base), cpb09-06 (25 cm above base), cpb09-07 (100 cm above base).

mas96-02. Dinner Creek ash. Gray ash, 2 m thick exposed along the north side of the Paulina-Suplee Highway, 17 km east of Paulina. 44.07090° N, 119.76753° W.

or99-01. Ash ~1.4 km east of Richland, OR on Hells Canyon Highway 89. 3 m massive gray vitric tuff in channel above basalt. Area characterized by abundant high angle normal faulting. 44.77243° N, 117.14386° W.
